# Fabrication of sensitive enzymatic biosensor based on multi-layered reduced graphene oxide added PtAu nanoparticles-modified hybrid electrode

**DOI:** 10.1371/journal.pone.0173553

**Published:** 2017-03-23

**Authors:** Md Faruk Hossain, Jae Y. Park

**Affiliations:** Department of Electronic Engineering, Kwangwoon University, Nowon Gu, Seoul, Korea; Institute of Materials Science, GERMANY

## Abstract

A highly sensitive amperometric glucose sensor was developed by immobilization of glucose oxidase (GOx) onto multi-layer reduced graphene oxide (MRGO) sheets decorated with platinum and gold flower-like nanoparticles (PtAuNPs) modified Au substrate electrode. The fabricated MRGO/PtAuNPs modified hybrid electrode demonstrated high electrocatalytic activities toward oxidation of H_2_O_2_, to which it had a wide linear response that ranged from 0.5 to 8 mM (R^2^ = 0.997), and high sensitivity of 506.25 μA/mMcm^2^. Furthermore, glucose oxidase-chitosan composite and cationic polydiallyldimethylammonium chloride (PDDA) were assembled by a casting method on the surface of MRGO/PtAuNPs modified electrode. This as-fabricated hybrid biosensor electrode exhibited high electrocatalytic activity for the detection of glucose in PBS. It demonstrated good analytical properties in terms of a low detection limit of 1 μM (signal-to-noise ratio of 3), short response time (3 s), high sensitivity (17.85 μA/mMcm^2^), and a wide linear range (0.01–8 mM) for glucose sensing. These results reveal that the newly developed sensing electrode offers great promise for new type enzymatic biosensor applications.

## Introduction

Effective and fast determination of glucose level is of substantial interest in many areas, including clinical diagnostics, bio and physiological research, as well as food companies [1, 2]. To date, many techniques, such as fluorescence, electrochemistry and flow injection, have been used to determine glucose concentration [3–5]. In particular, electrochemical enzyme-based biosensors are simple, reliable, quick, and sensitive [6]. In these sensor electrodes, the glucose concentration is indirectly determined by the measurement of a current related to the electrochemical detection of H_2_O_2_ [7]. In addition, the biopolymer chitosan is a type of matrix that is used for enzyme immobilization. It has fascinating properties that involve biocompatibility, good film forming ability, and high permeability toward water [8, 9]. On the other hand, polydiallyldimethylammonium chloride (PDDA) is a typical conducting polymer for the coating of developed electrodes that shows insignificant surface fouling and strong catalytic effects towards numerous biomolecules [10, 11]. PDDA has also been considered an excellent sensitizer and electron transfer mediator [12].

The oxidation of H_2_O_2_ can be promoted by bimetallic nanomaterials that have higher catalytic activity. Bimetallic nanomaterial assemblies are of particular interest in catalysis, since the second metal in the alloy/composite brings about variations in nanoparticle size, shape, surface structure, and physicochemical properties, including catalytic activity and chemical selectivity. In bimetallic nanoparticles (NPs), the secondary metal functions to oxidize the adsorbed intermediate poisons, or to suppress the adsorption of an intermediate poison [13], and higher electrocatalytic activity is therefore achieved with improved stability. Au is a comparatively less reactive metal and more electronegative than Pt, so Au—Pt alloy NPs can have unique effects on catalysis. Au is also important as a secondary element of AuPtNPs in a bimetallic alloy, as it oxidizes both the adsorbed poison intermediate and glucose [14]. Pt and Au nanoparticles could supply a suitable microenvironment for enzyme immobilization and facilitate electron transfer between the immobilized enzyme and PtNPs and AuNPs, therefore, they have been widely applied in biosensors. Furthermore, Pt and Au nanoparticles are very effective when used as a matrix for enzyme sensors since they are biocompatible and have a large surface area. Pt and Au nanoparticles have been widely used in enzymatic or nonenzymatic detection of biomolecules due to their outstanding catalytic activity for the redox reaction of hydrogen peroxide. The excellent catalytic activity of nanoparticles contributes to large surface area and suitable lattice plane which supply huge number of active centers for electrochemical reactions and make easier to the electron transfer reaction of hydrogen peroxide. Since enzymatic sensor is indirectly monitored by measurement of current associated with the electrochemical detection of hydrogen peroxide, consequently the sensor with high catalytic surface area shows high sensitivity. Metal alloy nanoparticles have mainly been obtained through chemical based methods, which include electrodeposition, chemical reduction, and pyrolysis. However, techniques of synthesized nanoparticles have a number of drawbacks, such as the requirements for elevated temperature, extensive template removal, and relatively high metal precursor [15]. Nevertheless, electrochemical synthesis for bimetallic nanoparticles is a simple approach to explore the application of nano structured hybrid materials in biosensor preparation.

Carbon based materials are widely used as catalytic support and have a potential for forming electrochemical sensing platform with high surface area [16]. However, graphene is a single or few layers of sp^2^- bonded carbon atoms with honeycomb lattice structure, and it has unique physicochemical properties, such as wide potential window, low charge resistance, and specific redox peaks [17]. Graphene oxide (GO) is a water-soluble colloidal suspension that is formed from the chemical reaction of graphite. The rough oxidations of crystalline graphite produce multi-layered GO, and it follows dispersion in aqueous medium through sonication or other processes [18]. Thus, the existence of oxygen-containing functional groups generates high defect density in oxidized graphene, thereby degrading its electrical, mechanical and thermal properties [19]. In general, chemical and thermal treatments reduce the oxygen containing functional group of GO to form reduced graphene oxide (RGO). The heterogeneous surfaces of RGO comprising edge plane nano bands are the only sites of electro-catalysis, whereas the basal plane is electro-chemically inert [20]. As the name suggests, single-layer RGO is several single layers of carbon atoms bonded together in a planar 2D platform. It is developed by repeated mechanical exfoliation [21], or by extremely controlled growth of single-layer graphene on substrates like SiC via chemical vapor deposition (CVD) [22]. Single-layer defects-free RGO is hard to develop in bulk. Furthermore, if the sheet surface of RGO becomes much more conductive, then RGO may be less inactive in chemical functionalization, due to the low concentration of reactive groups remaining on the sheet surface [23]. On the other hand, the presence of more surface oxygenated groups affects the resistivity of RGO [24]. Therefore, many efforts have been made towards the development of RGO using processes that include chemical, thermal, and electrochemical [25, 26]. Multi-layered reduced graphene oxide (MRGO) sheets can be found from the gentle exfoliation of RGO platelets that hold several layers, having an effective amount of oxygen functional groups. Thus, the high density of edge-plane defect sites on MRGO provides multiple active sites for electron transfer to biospecies [27].

In this work, multi-layered reduced graphene oxide (MRGO) was newly produced from the exfoliation of reduced graphene oxide platelets under moderate ultrasonication. An electrochemical process was then used to deposit bimetallic PtAu nanoparticles (NPs) onto the modified electrode. Glucose oxidase (GOx) was immobilized via entrapment using chitosan on a PtAuNPs decorated MRGO modified electrode surface, and PDDA was adsorbed on the GOx modified surface to inhibit enzyme leakage. Cyclic voltammograms and amperometric techniques were then used to characterize the proposed new glucose biosensor electrode for enzymatic glucose sensor applications. To the best of our knowledge, a similar preparation technique for MRGO, with nanostructured bimetallic hybrid electrode or enzyme preparation for glucose biosensing, has not been published elsewhere.

## Experimental

### Chemicals and apparatus

Chloroauric acid (HAuCl_4_, 99.9%), graphite powder (325 mesh size), hexachloroplatinic acid (H_2_PtCl_6_, 99.9%), ascorbic acid (AA, 99%), acetaminophen (AP, 99%), chitosan (Ch), 20% Poly(diallyldimethylammonium chloride) solution (PDDA), uric acid (UA, 99%), β-D(+) glucose (99.5%), chitosan (Ch, Mw 50,000–190,000) and glucose oxidase (GOx) (Aspergillus niger, VII type, lyophilized powder) were purchased from Sigma (Korea). The stock solution of glucose was diluted in phosphate buffered saline (PBS) solution, and high purity water (resistivity ≥ 18 MΩ-cm) was used as a solvent to prepare other solutions. Electrochemical analyses of the fabricated electrodes were conducted by electrochemical analyzer (Model 600D series, CH Instruments Inc., USA). In a three-electrode system, an Ag/AgCl electrode with 3 M sodium chloride and a flat Pt bar were utilized as the reference electrode (RE) and counter electrode (CE), respectively. The physical properties of the chemically modified graphite oxide (CGO), RGO pellets, MRGO sheet, and developed electrode were investigated by Fourier transform infrared spectroscopy (FTIR), X-ray photoelectron spectroscopy (XPS), Field emission scanning electron microscopy (FESEM) with Energy-dispersive X-ray spectroscopy (EDX), and Raman spectroscopy.

### Synthesis of CGO and RGO

Chemically modified graphite oxide (CGO) was synthesized using the modified Hummer methods [28, 29]. RGO was prepared using the following method. Briefly, 100 mg of CGO was dispersed into 60 mL of ethylene glycol, and the mixture was sonicated for 1.5 h at around 0–35°C. Afterward, 80 mL of water was dropped into the solution, followed by stirring for 1 h. Then, 540 mg of NaBH_4_ was carefully added, and the prepared solution was heated at 110°C for 2 h. After terminating the chemical reaction, the reaction product was filtered and washed several times with pure water. Finally, the filtered product was dried in a vacuum oven at 95°C overnight.

### Preparation of MRGO-modified gold electrode

Initially, titanium (Ti) of 35 nm and Au of 150 nm were sputtered on top of Si/SiO_2_ substrate, to make the nano-structured electrode. Then, RGO pellet was dispersed into a beaker with dimethylformamide (DMF) and pure water (1:1) solution, and followed by sonication at 180 W for 2.5 h at around 35°C. Next, 20 μL of the as-prepared RGO solution was cast onto the substrate electrode, and allowed to dry under room temperature. After being ambient dried, the MRGO-coated substrate electrode was washed with PBS and pure water, and then dried with N_2_ gas. The developed electrode was then put in a vacuum oven at 155°C for 12 h to eliminate the oxygen-containing functional groups and moisture, and to achieve good adhesion with the nanostructured surface.

### Preparation of MRGO/PtAuNPs/Ch-GOx/PDDA hybrid electrode

Ag/AgCl as a reference electrode, platinum sheet as a counter electrode, and the MRGO coated substrate electrode as the working electrode were used to deposit nanoparticles in the electrochemical system. The modification of MRGO with PtAuNPs was achieved by cyclic voltammogram under a potential range of -0.35 to +0.7 V in a precursor solution consisting of 5 mM of H_2_PtCl_6_, HAuCl_4_ (1:1), and 0.1 M KCl for 14 cycles. Then, pure water was used to clean the electrode, and N_2_ gas was used for drying the electrode. Similarly, different concentrated precursors (2.5 and 7.5 mM) with 0.1 M KCl were used to deposit the bimetallic nanoparticles. Next, 5 mg of glucose oxidase and 3 mg of chitosan were dissolved in 0.5 mL of pure water, then ultrasonicated for 5 min, and stirred until casting. Subsequently, 10 μL of the solution was cast onto the MRGO/PtAuNPs surface, and followed by drying at 4°C in a refrigerator. After drying the enzyme modified surface, 5 μL of 0.5% PDDA solution was cast onto the surface, and it was dried at 4°C in a refrigerator. The fabrication procedures for an enzymatic modified hybrid electrode, and a digital photograph of the fabricated hybrid electrode are showed in the supporting information (Fig A in S1 File).

## Results and discussion

### Physical characterization of MRGO and developed electrodes

**Fig 1:**
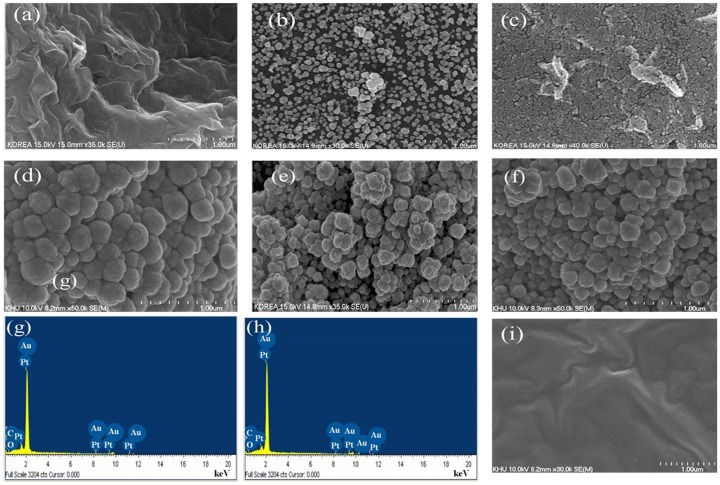
FESEM images of (a) MRGO, (b) MRGO/AuNPs, (c) MRGO/PtNPs, and (d) MRGO/PtAuNPs, for 2.5 mM precursor solution; (e) MRGO/PtAuNPs for 5 mM precursor solution; (f) MRGO/PtAuNPs for 7.5 mM precursor solution; corresponding EDX spectra of MRGO/PtAuNPs for (g) 2.5 mM loading nanoparticles, and (h) 5 mM loading nanoparticles; and (i) FESEM images of MRGO/PtAuNPs/Ch-GOx.

Fig 1 shows the surface morphology and elemental analysis of the as-developed MRGO. A FESEM image of MRGO formed after ultrasonication is shown in Fig 1a. A number of layers of MRGO sheet were found on the Au seed layer, and the crumpled structure of the MRGO sheets constructed during chemical reduction is evident. A crumpled or folded structure reduces the surface energy, and it also imparts a high Young’s modulus and good film-forming ability, due to nano-scale sheet interlocking [30]. However, MRGO creates a rougher surface due to the crumpled structure, which provides more active surface area for the decoration of the nanoparticles. The FESEM image (Fig 1a) clearly shows that the surface morphology is a suitable platform for biosensor preparation. The morphology of AuNPs and PtNPs are depicted in Fig 1b and 1c, respectively. These two figures show that no structural change occurs to the monometallic nanoparticles on the MRGO modified surface.

On the other hand, Fig 1d–1f (2.5, 5 and 7.5 mM) show that PtAu nanoflower-like structured nanoparticles cover the entire MRGO surface, making it significantly rougher, with more catalytic active sites and a larger active surface area. The flower-like structured nanoparticles were found to be due to the electrochemical co-deposition of bimetallic nanoparticles. This structure could be due to the secondary element during the formation of the hybrid nanoparticles [13]. It might form by the high-speed electrochemical deposition of PtAu nanoparticles on the MRGO modified surface in chloride concentrate media. Therefore, it demonstrates that secondary metal particles change the structure of the alloys nanoparticles. According to the surface roughness, which contributes to the flower-like structured particles shown in Fig 1e, the morphology of 5 mM concentrated PtAuNPs show a more favorable immobilized platform than those of other concentrated PtAuNPs. The optimal loading of bimetallic nanoparticles on the electrode surface might create a favorable platform. Fig 1g and 1h show the EDX analyses of elemental compositions of 2.5 and 5 mM loaded PtAuNPs modified electrodes, respectively. Noticeable peaks for carbon, oxygen, platinum, and gold were investigated for MRGO/PtAuNPs. For the 2.5 mM concentration precursors, the weight percentages of carbon, oxygen, platinum, and gold were 14.23%, 0.87%, 29.46%, and 55.46%, respectively; and for the 5 mM precursors, the percentages of carbon, oxygen, platinum, and gold were 5.95% 0.30%, 33.54%, and 60.21%, respectively. The data demonstrated that due to the electrostatic interaction from the negatively charged MRGO sheets, a greater weight percentage of Au compared to Pt particles accumulated on the electrode surface. This is probably because the Au occupied three valence electrons in the precursor, whereas Pt occupied four valence electrons. Therefore, chloride concentrated media generated Au^3+^ faster than Pt^4+^. These accumulated ions would then reduce to generate the PtAu nanoparticles as a nanoflower structure [31]. As a result, more Au particles were deposited on the surface than Pt particles. This indicates that the PtAuNPs were successfully deposited under the given conditions, and donated equally towards the construction of the modified electrode for biosensing. A chitosan and GOx solution was cast onto the MRGO/PtAuNPs surface as shown in Fig 1i. We can see that glucose oxidase well dispersed in chitosan and adsorb on the surface via entrapment. The biosensor materials are fixed on the surface of the modified electrode.

**Fig 2:**
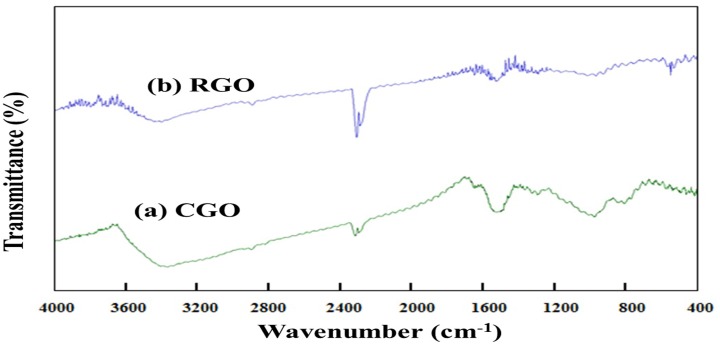
FTIR spectra of (a) chemically modified graphite oxide (CGO), and (b) reduced graphene oxide (RGO) powder.

CGO powder and RGO pellets powder were analyzed by FTIR spectroscopy. These spectra indicate the availability of different types of bonds in the CGO and RGO as shown in Fig 2. Several signature peaks of CGO were generated at 3413.0, 1721.3, 1618.07, 1379.2, and 1071.38 cm^-1^. The peak at 1379.2 cm^-1^ is due to the distortion vibration of—OH, whereas the peak at 1071.38 cm^-1^ is the C-O stretching vibration of alkoxy groups [25, 32].

The peak at 1618.07 cm^-1^ relates to the O—H bending vibration of the absorbed water molecules and the vibration of aromatic C = C; the peak at 1721.3 cm^-1^ comes from C = O stretching vibration; and the 3413.0 cm^-1^ originates from the—OH stretching vibration. In contrast, the main peaks of the RGO pellets become visible at 3425.32, 1725.07, 1640.2, 1571.76, and 1427.47 cm^-1^. The peak at 1427.47 cm^-1^ originates from the distortion vibration of -OH. The indicated peaks at 1725.07 and 3425.32 cm^-1^ are due to the -OH stretching vibration and the C = O stretching vibration, respectively. Moreover, the peak at 1577.7 cm^-1^ for the RGO is due to the C = C skeletal vibration of graphene sheets [33]. A comparison between Fig 2a and 2b shows that most of the functional peaks were significantly reduced, which confirms the successful reduction of graphene oxide. The chemical treatment of the CGO decomposed the oxygen containing functional groups. Therefore during the reduction reaction of CGO, the concentration of the oxygen functional groups was minimized.

**Fig 3:**
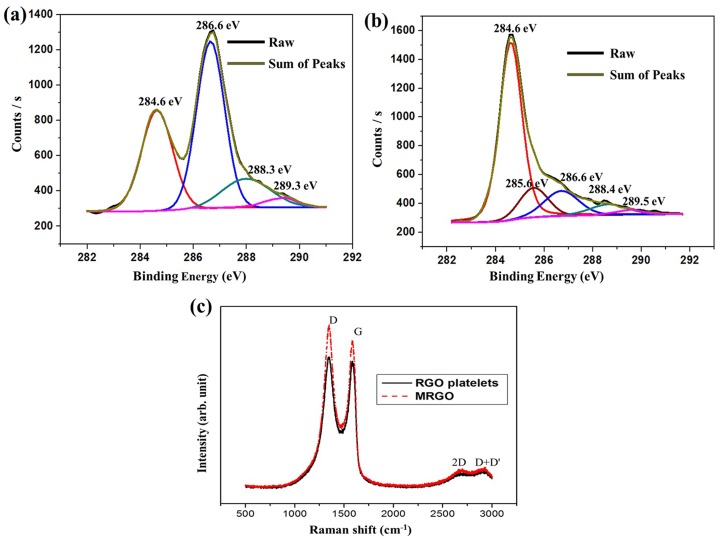
XPS spectra of (a) CGO, and (b) MRGO; and (c) Raman spectra of RGO platelets (solid line), and MRGO sheet (dotted line).

The C 1s XPS spectra of CGO powder and MRGO sheets are shown Fig 3a and 3b, respectively. These spectra clearly suggest a considerable degree of oxidation that correlates with the carbon atoms in various functional groups: the non-oxygenated ring C (284.6 eV) that covers the C = C bond of hybridized sp^2^, the C in C—O bonds (286.6 eV) that involve the hydroxyl and epoxy groups, the C in C = O bonds (288.4 eV) that involve the carbonyl groups, and the C in OH—C = O (289.3) bonds that involve carboxyl groups [34]. The reduction of CGO generates a new peak (285.6 eV); it is sp^3^ hybridized, and involved in the C-C bond. The spectra analysis shows that the chemical reduction process and heat treatment on the substrate remarkably reduced the oxygen containing functional groups of CGO. XPS was also performed to study the composition of the PtAu bimetallic nanoparticles on the MRGO modified electrode (Fig B in S1 File). The atomic percentages of elements in MRGO/PtAuNPs for 5 mM precursors are presented in the supporting information (Table A in S1 File).

Fig 3c shows the Raman spectra of RGO pellets (solid line) and MRGO sheets (dotted line). Raman spectroscopy is an effective tool for the detection of the single and/or multilayer phenomena of graphene sheets. The two defined peaks, named D—band and G—band, of the GO appear at 1349 and 1588 cm^-1^, respectively. Similarly, the MRGO peaks appear at 1347 and 1595 cm^-1^. The G—band is involved in the in-plane vibration of sp^2^ bonded carbon atoms, whereas the D—band is related to the vibrations of carbon atoms with the sp^3^ electronic arrangement of disordered graphene.

The multi-layer and the position of the 2D band are known as the key parameters for the identification of the number of layers of graphene sheets or reduced graphene oxide sheets [35, 36]. In this work, the presence of the 2D band in the Raman spectra of the RGO pellets and MRGO film at around 2694 cm^-1^ indicated the presence of multilayer RGO pellets and multilayer RGO sheets [35], as FESEM image analysis shows.

### Electrochemical performance of MRGO/PtAuNPs modified hybrid electrode towards H_2_O_2_

**Fig 4:**
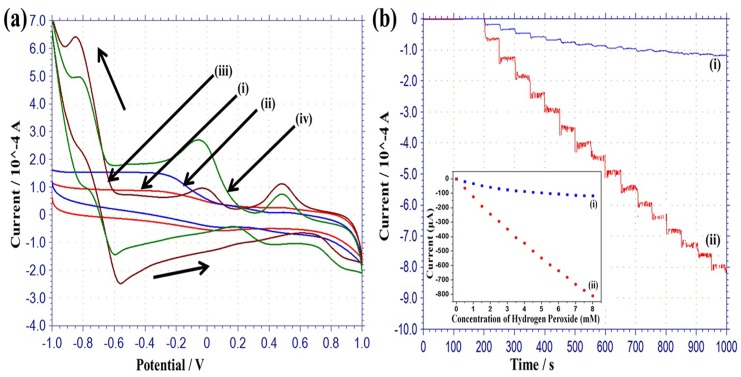
(a) Cyclic voltammograms (CVs) of fabricated MRGO modified electrodes (i) in PBS solution, (ii) in 2 mM hydrogen peroxide solution; and MRGO/PtAuNPs modified hybrid electrode (iii) in PBS, and (iv) in 2 mM hydrogen peroxide solution (PBS solution pH 7. 4, 50 mM), scan rate: 50 mV/s. (b) Amperometric response of (i) MRGO, and (ii) MRGO/PtAuNPs modified hybrid electrodes in PBS, to the consecutive addition of the concentration of H_2_O_2_ in 0.5 mM at 0.6 V, with inset calibration plots.

The cyclic voltammogram (CV) of a typical MRGO modified electrode was conducted in 0.05 M PBS as shown in Fig 4a. A wide oxidation peak (-0.15 V to 0.3 V) and a wide reduction peak (0.0 V to -0.4) are evident. The redox peaks are ascribed to the redox pair of some electrochemically active oxygen-containing groups on the MRGO planes that are too stable to be reduced oxygen containing functional groups by chemical and thermal processes [37]. The electrochemically active oxygen functional groups are carbonyl and carboxyl groups, which have limited influence on the conductivity of MRGO sheet. Fig 4a(ii) shows the CV of the MRGO modified electrode in 2 mM H_2_O_2_ solution. It is apparent that the anodic current of the MRGO modified electrode in H_2_O_2_ solution is higher by from 0.4 to 1 V than that of the MRGO modified electrode in PBS solution. In contrast, the cathodic current of the MGRO modified electrode in 2 mM H_2_O_2_ solution is smaller by from 1 to 0.25 V than that of the MGRO modified electrode in PBS solution. This result indicates that the catalytic performance of MRGO modified electrode increases in the positive potentials, due to H_2_O_2_ oxidation. Fig 4a(iii) exhibits the CV of the MRGO/PtAuNPs modified hybrid electrode preparation from the 5 mM precursor solution. In this figure, broad peaks and weak peaks appear, with oxidation peaks at around -0.80 and -0.58 V, and reduction peaks at around 0.5, 0.0 and -0.85 V due to the action of the redox reaction of Pt and Au nanoparticles. The peak at -0.58 V on the oxidation curve is for Pt, which created PtOH [38], while the peak at -0.80 V is due to the adsorption of H on the surface of the PtAuNPs. The peak at -0.85 V on the reduction curve should occur due to the desorption of H on the surface of the PtAuNPs. The cathodic peak at 0.0 V is due to the formation of oxide reduction of Pt nanoparticles, while the peak at 0.5 V corresponds to the reduction of Au oxide. These results are due to the bimetallic nature of the Pt and Au nanoparticles. The potential redox reaction may be described by the following equations:

$$Pt+H_{2}O=PtOH+H^{+}+e^{-}$$ (1)

$$PtOH=PtO+H^{+}+e^{-}$$ (2)

$$Au+H_{2}O=AuOH+H^{+}+e^{-}$$ (3)

$$AuOH=AuO+H^{+}+e^{-}$$ (4)

The CV of the MRGO/PtAuNPs-modified hybrid electrode in the 2 mM H_2_O_2_ solution is shown Fig 4a(iv). Fig 4a(iii) and 4a(iv) observe that the oxidation current (from 0.4 to 1 V) of the MRGO/PtAuNPs in H_2_O_2_ with PBS is higher than that of the MRGO/PtAuNPs in PBS. In contrast, the reduction current (from 1 to 0.25 V) of the MRGO/PtAuNPs in H_2_O_2_ with PBS is lower than that of the MRGO/PtAuNPs in PBS. This result indicates that due to oxidation, MRGO/PtAuNPs offers good electrocatalytic activity in H_2_O_2_ solution in the positive potentials. Moreover, 2.5 and 7.5 mM loaded PtAuNPs modified MRGO electrodes were also conducted in the CVs in PBS with a 2 mM H_2_O_2_ solution, which are not shown in the manuscript. It was found that 5 mM loaded PtAuNPs based electrode demonstrated a higher oxidation current than that of the 2.5 and 7.5 mM loaded PtAuNPs based electrodes. Therefore in this work, the 5 mM concentrated PtAuNPs based electrode was investigated in more detail.

The amperometric responses of fabricated electrodes detected under different amounts of H_2_O_2_ concentration at a predetermined potential are an important factor for the assessment of an amperometric sensor as shown in Fig 4b. The amperometric responses of MRGO and MRGO/PtAuNPs modified hybrid electrodes, respectively, at +0.6 V upon consecutive drops of H_2_O_2_ in PBS solution, together with the calibration curves inserted in the inset of the figures as shown Fig 4b(i) and 4b(ii). This potential was chosen because of the higher sensitivity and linear range obtained for glucose detection, as shown in the subsequent section. The response of the MRGO modified electrode is weak; whereas, the response of the MRGO/PtAuNPs modified hybrid electrode severely increases after each injection of H_2_O_2_, representing the effective recognition of H_2_O_2_ catalyzed by PtAu bimetallic nanoparticles. The response time of the MRGO/PtAuNPs modified hybrid electrode is 2 s, with linear detection range from 0.5 to 8 mM, and a sensitivity of 506.25 μA/mMcm^2^ at 0.6 V. This amperometric response reveals that the bimetallic particles exhibit drastic electrocatalytic activity on the PtAuNPs-modified electrode, which is subjected to the higher specific surface area of bimetallic particles (PtAuNPs). The high active surface area of MRGO supplies a huge number of anchoring sites for the decoration of PtAuNPs, and the modified electrode contributes to the synergistic effect of MRGO and the bimetallic nanoparticles.

### Electrocatalytic characterization of MRGO/PtAuNPs/Ch-GOx/PDDA electrode

**Fig 5:**
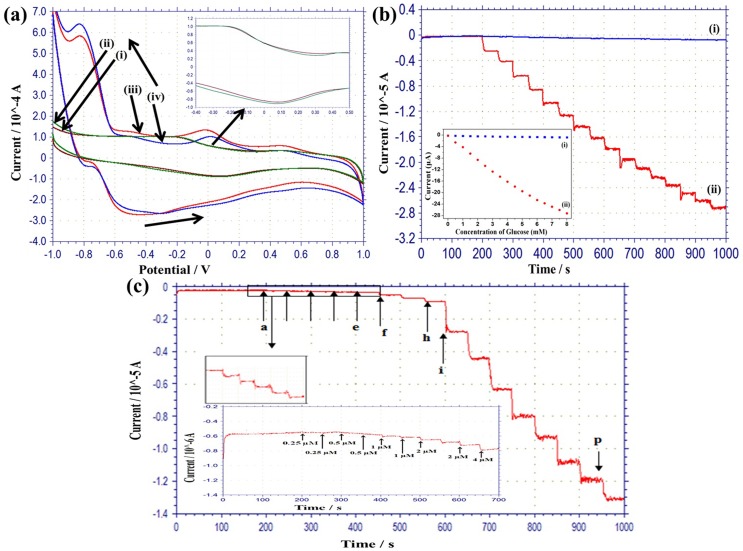
(a) CVs of fabricated hybrid MRGO/Ch-GOx/PDDA electrodes (i) in PBS solution, and (ii) in 2 mM glucose; and MRGO/PtAuNPs/Ch-GOx/PDDA (iii) in PBS, and (iv) in 2 mM glucose, (close up views of MRGO/Ch-GOx/PDDA modified electrode in PBS and PBS with glucose solution). (b) Amperometric response of (i) MRGO/Ch-GOx/PDDA and (ii) MRGO/PtAuNPs/Ch-GOx/PDDA modified electrode in PBS (pH, 7.4, 50 mM) to consecutive drops of concentration of glucose in 0.5 mM at 0.6 V, with inset calibration plots. (c) Amperometric response of MRGO/PtAuNPs/Ch-GOx/PDDA modified hybrid electrode in PBS to consecutive drops of different concentration (a-e) 10 μM, (f-h) 50 μM, and (i-p) 0.5 mM of glucose at 0.6 V; inset shows low detection limit of glucose.

Fig 5a shows cyclic voltammograms (CVs) of the typical enzyme modified hybrid electrodes in 0.05 M PBS. The CV of the MRGO/Ch-GOx/PDDA modified hybrid electrode in PBS solution is shown in Fig 5a(i). The inset of Fig 5a shows a peak in the cathodic direction at around 0.45 V. This peak might be ascribed to the absorption of poison intermediates. Fig 5a(ii) observes that when 2 mM glucose is injected in the PBS, the anodic background current (-0.4V to 0.4V) increases, but the cathodic background current (0.4 to 0.1 V) decreases during positive and negative sweep. This result indicates that the oxidation current can increase due to the resulting adsorption of OH on the surface of the MRGO/Ch-GOx/PDDA modified biosensor electrode. When the enzyme loaded electrode is immersed in a glucose containing solution, glucose is oxidized by the catalytic action of enzyme and produced gluconic acid and H_2_O_2_. Then, the free hydrogen peroxide decomposes to generate oxygen and proton, and the reversible reaction occurs on the electrode surface during CV [39]. The probable mechanism is described as,

$$Glu\text{cos}e+O_{2}=\begin{array}{cc} Gluconic & acid+ \end{array}H_{2}O_{2}$$ (5)

$$H_{2}O_{2}=O_{2}+2H^{+}+2e^{-}$$ (6)

$$H_{2}O_{2}+2e^{-}=2OH^{-}$$ (7)

$$MRGO+OH^{-}=MRGO(OH)+e^{-}$$ (8)

$$MRGO(OH)+H^{+}+e^{-}=MRGO+H_{2}O$$ (9)

The CVs of the MRGO/PtAuNPs/Ch-GOx/PDDA modified electrode in the PBS solution present in Fig 5a(iii). Fig 5a(iii) and 5a(iv) show that the background reduction current in the range from 0.09 to -0.6 V in the 2 mM glucose is lower than that in the glucose free PBS, and that the oxidation current from -0.2 to 1.0 V in the 2 mM glucose is higher than that in glucose free PBS. This is probably because the oxidation current increases the amount of adsorbed OH on the surface of the MRGO/PtAuNPs/Ch-GOx/PDDA modified electrode. Moreover, the PtAuNPs-modified electrode has the ability to protect intermediates poisons, due to the novel metal particles used [40]. This outcome indicates that the MRGO/PtAuNPs/Ch-GOx/PDDA has good electrocatalytic activity through glucose oxidation.

The amperometric responses of the MRGO/Ch-GOx/PDDA modified electrode at 0.6 V upon continuous drops of 0.5 mM glucose in PBS solution as shown in Fig 5b(i), and the inset of this figure shows the respective calibration curve. The calibration curve shows linear detection from 0.5 to 8 mM with sensitivity of 0.394 μA/mMcm^2^. It shows a poor signal after consecutive injection of glucose at 0.6 V due to the absence of nanoparticles. These results reveal that the catalytic activity of the MRGO/Ch-GOx/PDDA modified electrode is not so much in H_2_O_2_ at the potential of 0.6 V.

Amperometric measurement of the MRGO/PtAuNPs/Ch-GOx/PDDA- modified hybrid electrode was carried out at 0.6 V. Amperometric detection of the glucose was performed at 0.6 V with consecutive drops of glucose (0.5 mM) in Fig 5b(ii), while the inset of this figure shows the corresponding calibration curve for the respective electrode. The biosensing electrode showed a linear detection range of 0.5–8 mM with a sensitivity of 17.85 μA/mMcm^2^ (R^2^ = 0.993) at 0.6 V. Fig 5c shows the various concentrations of glucose measured using the fabricated hybrid electrode at 0.6 V in PBS, and the inset of Fig 5c shows the detection limit of glucose was determined to be 1 μM (the signal is three times greater than the noise). Table 1 compares various glucose sensing electrodes with respect to sensitivity, detection range, and detection limit. The table shows that in comparison to the represented glucose biosensor electrodes [41–49], the performance of the developed sensor electrode is good.

**Table 1:** Performance of different glucose-sensing hybrid electrodes.

Sample	Linear range	Detection limit	Sensitivity	References
GCE/CMM-PtNPs/ GOx	0–4.2 mM	0.001 mM	0.962 μA/mM	[41]
ITO/ZnO/AuNP/GOx	2.78–22.2 mM		3.12 μA/mMcm^2^	[42]
GCE/NH_2_-PB-GO-HPts/GOx	0.01–5.23 mM	0.0033 mM	-	[43]
GCE/CNT/Au/PDDA/GOx	0.5–5 mM	-	3.96 μA/mMcm^2^	[44]
GCE/Gr/CdS/GOx	2–16 mM	0.7 mM	1.76 μA/mMcm^2^	[45]
Pt/TiO_2_/RGO/PtNPs/ GOx	0.1–8 mM	0.1 mM	0.94 μA/mMcm^2^	[46]
GCE/IL-Gr/ AuNPs/GOx	2–20 mM	0.13 mM	0.16 μA/mM	[47]
PET/Ti/Au/SDS-MWCNT/PDDA/GOx/PDDA	0.02–2.2	0.01 mM	5.6 μA/mMcm^2^	[48]
Pt/PtZn/GOx/PEDOT-PDDA	0.2–2.5 mM	-	13.5 μA/mMcm^2^	[49]
Au/MRGO/PtAuNFs/Ch-GOx/PDDA	0.01–8 mM	0.001 mM	17.85 μA/mMcm^2^	This work

### Interference effect, reproducibility, recovery and blood plasma test on biosensor

**Fig 6:**
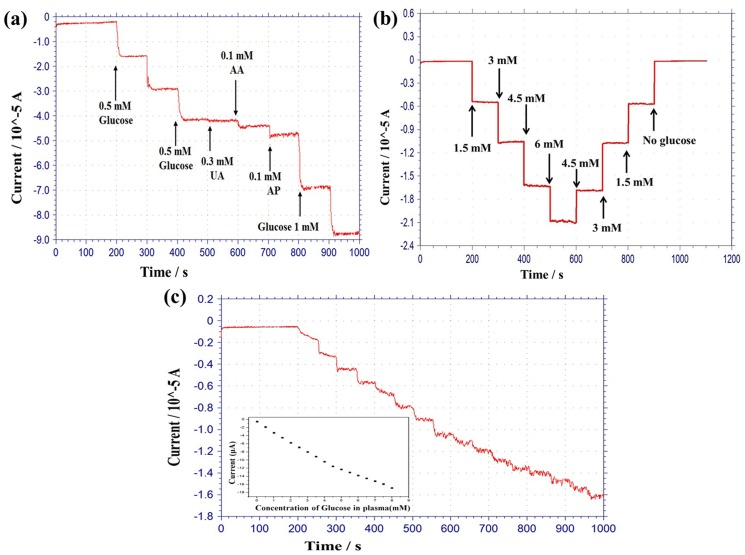
(a) The current response of interference species on the MRGO/PtAuNPs/Ch-GOx/PDDA hybrid electrode upon addition of 1.5 mM glucose, then 0.3 mM UA, 0.1 mM AP, 0.1 mM AA, and further drops of 1 mM glucose in PBS solution at 0.6 V. (b) Current—time recordings for recovery characteristic of MRGO/PtAuNPs/Ch-GOx/PDDA modified electrode in step-change of glucose concentration. (c) Amperometric responses of MRGO/PtAuNPs/Ch-GOx/PDDA biosensor in PBS solution for consecutive addition of glucose in human blood plasma.

Anti-interference characteristics are also significant factors for sensing applications. The interferences from electroactive species generally present in physiological samples of glucose, such as uric acid (UA), acetaminophen (AP), and ascorbic acid (AA), have previously caused problems in the proper determination of glucose. Actually, a low concentration of interference is applied, because the average concentration in blood of AP is 0.066–0.2 mM, of AA is 0.05–0.143 mM, and of uric acid is 0.09–0.48 mM [7, 50]. The response of interfering oxidation currents were less than the 2.75 μA value of 1 mM glucose. The current responses of 0.18 μA for 0.1 mM AA (this oxidation current is 15 times less than that of 1 mM glucose), negligible current for 0.3 mM UA, and 0.32 μA for 0.1 mM AP (this oxidation current is 9 times less than that of 1 mM glucose) at 0.6 V as shown Fig 6a.

Four glucose-sensing electrodes were fabricated using the same process as that used for the respective electrode. All the electrodes were measured in continuous additions of 0.5 mM glucose up to 5 mM in PBS solution. All different biosensor electrodes exhibit standard deviations of 9.1% in amperometric response. This indicates that the fabricated sensing electrodes had an acceptable reproducibility with respect to amperometric measurement. “Repeatability of the fabricated sensor was performed by amperometric response in PBS up to 5 mM glucose. The glucose sensor showed a relative standard deviation (RSD) of 4.3% for five repetitive measurements. It is indicated that the fabricated sensor had a good repeatability. The stability of the developed electrode was also investigated for four weeks. During that time, the sensitivity of the respective sensing electrode was decreased by 17.65%. The results obtained reveal that the sensor electrode has long-term stability. Fig 6b shows a current-time recording for the recovery characteristics of MRGO/PtAuNPs/Ch-GOx/PDDA-modified hybrid bioelectrode towards stepwise changes in the glucose concentration. This test was conducted on different samples of glucose (no glucose, 1.5, 3, 4.5, and 6 mM). The total measurements were executed with a stepwise change of glucose concentration with 100 s regular intervals. The figure shows the current response was very stable towards both increments and decrements of the glucose concentration. The recovery rates for the no glucose state, 1.5, 3, and 4.5 mM were 100.5, 100.3, 100.9, and 102.87%, respectively.

In order to evaluate the performance of the MRGO/PtAuNPs/Ch-GOx/PDDA sensing electrode, glucose in biofluidic solution was also investigated. The amperometric technique was conducted to detect glucose with consecutive drop of 0.5 mM glucose of blood plasma in PBS solution. Fig 6c presents the current response of the as-fabricated glucose-sensing electrode. This shows a stable and quick current response (0.5-8mM) at biofluidic solutions. However, the sensitivity of the hybrid electrode decreased by about 28% when the electrode measured the glucose of human plasma. Similar reductions of biosensor sensitivity have also been reported [7, 51]. This reduction may be due to the adsorption of various proteins and cells on the sensor surface during the measurement of glucose of blood plasma. In addition, the relative standard deviation (RSD) of the current response to 4 mM glucose of blood plasma was 6.38% for 5 consecutive detections.

## Conclusions

Multi-layered reduced graphene oxide (MRGO) was successfully developed by the treatment of CGO, and an MRGO/PtAu nanoflower-like structured nanoparticle-modified hybrid electrode was fabricated. This modified hybrid electrode showed higher electrocatalytic activity towards the oxidation of H_2_O_2_. It demonstrated good analytical activities, with a short response time of 2 s, sensitivity of 506.25 μA/mMcm^-2^, and linear range of 0.5–8 mM towards oxidation of H_2_O_2_. Moreover, after the immobilization of Ch-GOx composite integrated with PDDA on the surface of MRGO/PtAuNPs, a novel and biocompatible glucose-sensing electrode was successfully constructed. The hybrid electrode exhibited high catalytic activity toward glucose oxidation, with sensitivity of 17.85 μA/mMcm^2^, response time of 3 s, and linear range of 0.01–8 mM for glucose detection. This sensor electrode also showed an acceptable interference effect, reproducibility, and stability. For practical assessment, the as-prepared sensor electrode was used to measure the glucose of a blood plasma solution, and showed a wide linear range of glucose plasma detection. The results obtained with the electrode led to the successful construction of a practical glucose biosensor and fuel cell, which could also be used for the immobilization of some other biomolecules.

## Supporting information

S1 File (DOCX)
