# Supplementary material for: Fabrication of sensitive enzymatic biosensor based on multi-layered reduced graphene oxide added PtAu nanoparticles-modified hybrid electrode
Source: PLoS One. 2017 Mar 23;12(3):e0173553. doi: 10.1371/journal.pone.0173553 (PMC5363929; doi:10.1371/journal.pone.0173553)
Supplement: S1 File — (DOCX) [file pone.0173553.s001.docx]

**Supporting information**

**Fabrication of Sensitive Enzymatic Biosensor Based On Multi-Layered Reduced Graphene Oxide Added PtAu Nanoparticles-Modified Hybrid Electrode**

Md Faruk Hossain, Jae Y. Park^[[1]](#footnote-1)^*

Department of Electronic Engineering, Kwangwoon University, Nowon Gu, Seoul, Korea

**
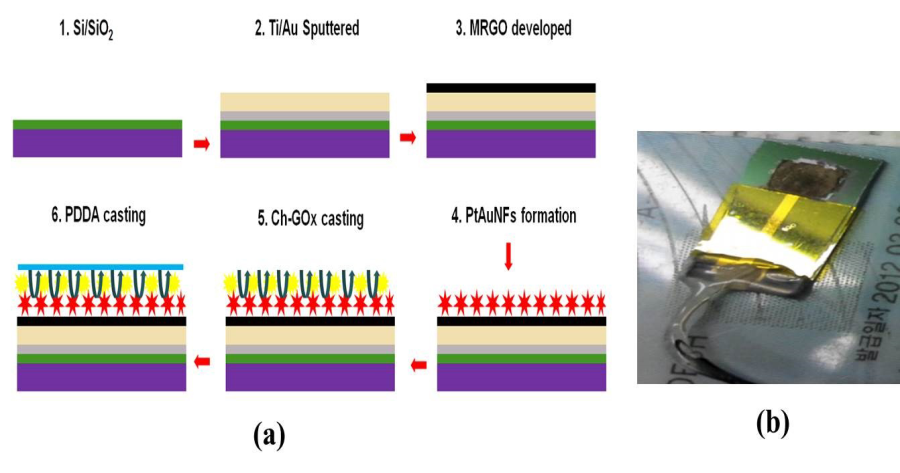
**

Fig A in S1 File. (a) Fabrication procedures of hybrid biosensor electrode, and (b) a digital photograph of the fabricated hybrid sensor electrode.

**
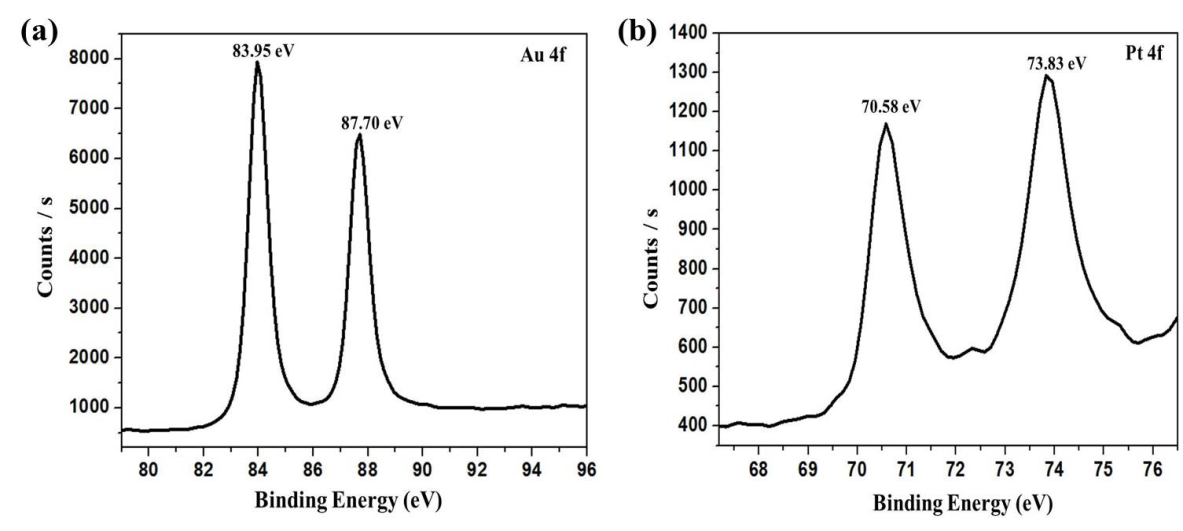
**

Fig B in S1 File. XPS spectra of (a) Au nanoparticles on MRGO/PtAuNPs, and (b) Pt nanoparticles on MRGO/PtAuNPs.

Fig Aa in S1 File illustrates the fabrication procedures and Fig Ab in S1 File shows a digital photograph of the fabricated hybrid electrode.

The XPS spectrum of Au 4f and Pt 3f core for the region of MRGO/PtAuNPs modified electrode are shown in Figs Ba and Bb in S1 File, respectively. The Au 4f core level spectrum for the AuNPs presents main peaks at 83.84 and 87.62 eV, which corresponds to the binding energies of Au 4f7/2 and Au 4f5/2, respectively. The Pt 4f core level has two peaks for 4f7/2 and 4f5/2 with binding energies at 70.58 and 73.83 eV, respectively. Compared to the standard data of the Pt^0^ (70.9 and 74.2 eV) and Au^0^ (84.0 and 87.7 eV) species [1], the binding energies of both Pt 4f and Au 4f are slightly shifted to lower values. The decrease in the binding energies indicates that electron transfer can occur from the reduced graphene oxide sheet to the PtAuNPs. The results are in good agreement with previous reports [1-3] and this further confirms that the PtPd bimetallic nanoparticles have been successfully deposited under the given condition on the MRGO modified electrode. The XPS analysis was used to obtain the composition of elements in the MRGO/PtAuNPs. The atomic percentages of elements in MRGO/PtAuNPs for 5 mM precursors are presented in Table A in S1 File.

Table A in S1 File. Atomic percentages of various elements in MRGO/PtAuNPs.

| **Matrix** | **C 1s** | **O 1s** | **Pt 4f** | **Au 4f** |
| --- | --- | --- | --- | --- |
| MRGO/PtAuNPs | 48.25 | 11.58 | 14.31 | 25.86 |

**References**

1. You HJ, Zhang FL, Liu Z, Fang JX (2014) Free-standing Pt–Au hollow nanourchins with enhanced activity and stability for catalytic methanol oxidation. ACS Catal 4: 2829-2835
2. Li J, Liu CY, Liu Y (2012) Au/graphene hydrogel: synthesis, characterization and its use for catalytic reduction of 4-nitrophenol. J Mater Chem 22: 8426-8430
3. Xiong Z, Zhang LL, Ma J, Zhao XS (2010) Photocatalytic degradation of dyes over graphene–gold nanocomposites under visible light irradiation. Chem Commun 46: 6099-6101

1. * Corresponding author. Tel.: +82-2-940-5604; fax: +82-2-942-1502;

   e-mail: [jaepark@kw.ac.kr](mailto:jaepark@kw.ac.kr), [faruk.kwu@hotmail.com](mailto:faruk.kwu@hotmail.com) [↑](#footnote-ref-1)
